# A new parameter in predicting contrast-induced nephropathy: Osaka prognostic score

**DOI:** 10.1590/1806-9282.20240423

**Published:** 2024-08-16

**Authors:** Nail Burak Özbeyaz, Engin Algül

**Affiliations:** 1 Ankara University, Faculty of Medicine, Department of Cardiology – Ankara, Turkey.; 2 University of Health Sciences, Etlik City Hospital, Department of Cardiology – Ankara, Turkey.

**Keywords:** Osaka prognostic score, Inflammation, Nutrition, Contrast-induced nephropathy, Acute coronary syndrome, Percutaneous coronary intervention

## Abstract

**OBJECTIVE:**

Nowadays, the frequency of complications is also increasing following the increasing frequency of coronary angiography and percutaneous coronary intervention. Contrast-induced nephropathy is one of the most common of these complications. This study aimed to investigate the relationship between the Osaka prognostic score, which has previously been shown to have prognostic importance in gastrointestinal malignancies, and the development of contrast-induced nephropathy.

**METHODS:**

The study retrospectively examined the data of 1,498 patients who underwent coronary angiography and percutaneous coronary intervention due to acute coronary syndrome between 2018 and 2023. Demographic characteristics and laboratory findings were retrospectively collected from patients’ charts and electronic medical records.

**RESULTS:**

Osaka prognostic score (0.84±0.25 vs. 2.2±0.32, p<0.001) was higher in patients who developed contrast-induced nephropathy. Also, Osaka prognostic score [OR 2.161 95%CI (1.101–4.241), p<0.001] was found to be an independent risk factor along with age, diabetes mellitus, systolic pulmonary artery pressure, hemoglobin, hemoglobin, C-reactive protein, albumin, N-terminal brain natriuretic peptide, and systemic immune-inflammation index. The receiver operating characteristic curve showed that the optimal cutoff value of Osaka prognostic score to predict the development of contrast-induced nephropathy was 1.5, with a sensitivity of 83.4 and a specificity of 65.9% [area under the curve: 0.874 (95%CI: 0.850–0.897, p≤0.001)].

**CONCLUSION:**

Osaka prognostic score may be an easily calculable, user-friendly, and useful parameter to predict the development of contrast-induced nephropathy in patients undergoing percutaneous coronary intervention after acute coronary syndromes.

## INTRODUCTION

Acute coronary syndromes (ACS) are a common cause of mortality and morbidity today. Especially with increasing life expectancy, the frequency of ACS also increases^1^. Today's gold standard ACS diagnosis and treatment protocol is coronary angiography (CAG) and percutaneous coronary intervention (PCI)^2^. With the increasing frequency of ACS, the number of PCIs performed is also increasing. Although successful coronary revascularization has been achieved, contrast-induced nephropathy (CIN), which may occur in association with PCI, increases mortality and morbidity in patients and prolongs hospitalization, which can lead to poor outcomes^3^. Classically, CIN is defined as an increase in serum creatinine value after PCI by 5 mg/dL or more than 25% within 48–72 h^4^.

The etiopathogenesis of CIN has a multicomponent structure. Although direct contrast agent-related renal cytotoxicity plays the leading role, local ischemia related to renal hypoperfusion (caused by decreased cardiac output), excessive activation of the immune system, and endogenous vasomotor imbalances that may occur as a result of ACS also contribute to the formation of CIN^5,6^. It is also known that poor nutritional status is directly related to CIN^7,8^. Osaka prognostic score (OPS) is a new marker based on inflammation and nutrition that has emerged recently and has been reported to have prognostic importance in gastrointestinal malignancies. OPS includes C-reactive protein (CRP), albumin, and lymphocyte count (TLC)^9^. It is known that all of these components are individual risk factors for CIN^10,11^. When all this information is evaluated, it is observed that low nutritional status and the presence of high inflammation increase CIN. OPS provides information on both parameters. For this reason, our study aimed to investigate the relationship between OPS and CIN that develops after PCI, which has not been investigated before.

## METHODS

### Population

This study retrospectively examined the data of 1,498 patients admitted to the hospital with ACS and underwent PCI within 24 h between September 2018 and December 2023. The study was started after receiving approval from the local ethics committee. All steps of the study were planned and carried out following the directives of the Declaration of Helsinki. Patients with active infection, end-stage renal failure, use of nephrotoxic drugs in the last week, and use of contrast material for another reason in the last week, patients whose data could not be accessed for any reason, and patients with cardiogenic shock and any malignancy were excluded from the study. Patients with ST-elevation myocardial infarction (STEMI) were urgently taken immediately after diagnosis. Patients with non-STEMI (NSTEMI) were taken for CAG, and PCI was performed within 24 h at the latest. A nephrotropic, water-soluble, low osmolar, non-ionic contrast agent, Iohexol (300 mg iodine/mL; 672 mosml/kg of water; Omnipaque; GE Healthcare Inc., Marlborough, MA, USA) was used for angiography. Whole blood parameters were obtained from automatic hematology analysers (Symex XN-550 analyzer, Symex, Kobe, Japan), and biochemical data were obtained from biochemistry devices (Beckman Coulter Inc., Brea, New York, USA).

### Definitions

Three variables are used to calculate the OPS: CRP (≤10.0 mg/L: 0 point and>10.0 mg/L: 1 point), albumin (≥3.5 g/dL: 0 point and<3.5 g/dL: 1 point), and TLC (≥1,600/μL: 0 point and<1,600/μL: 1 point). OPS was calculated as the sum of the scores from these parameters, thus resulting in four OPS-based groups: four groups (zero, one, two, and three). For Glasgow prognostic score (GPS), CRP (≤10.0 mg/L: 0 points and>10.0 mg/L: 1 point) and albumin values (≥3.5 g/dL: 0 points and<3.5 g/dL: 1 point) were used. Patients were grouped according to the scores from these parameters by receiving 0, 1, and 2 points^9^. The formula 10×albumin (g/dL)+0.005× TLC (per mm^3^) was used to calculate the prognostic nutritional index (PNI)^12^, and the formula platelet count*neutrophil count/lymphocyte count was used to calculate the systemic immune-inflammatory index^13^.

### Statistical analysis

Categorical data are presented as numbers and percentages. For non-parametric data analysis, the chi-square test was used. All the variables obtained were examined with the Kolmogorov-Smirnov test for normality and the Levene test for homogeneity of variances before the significance tests were used. Normally distributed homogeneous data were evaluated with a t-test in independent groups and a Mann-Whitney U test for results that did not show normal distribution. Receiver operating characteristic (ROC) analysis was used to estimate the optimal cutoff value of OPS, GPS, and systemic immune-inflammation index (SII) in indicating CIN. Sensitivity, specificity, and area under the curve (AUC) were calculated. Logistic regression analysis was performed to determine values predicting CIN. The values that differed among these parameters for CIN were included in the univariate logistic regression analysis, and their significance was determined. Potential risk indicator parameters that were significant in the univariate logistic regression model were included in the multivariate logistic regression analysis (forced entry method). The analyses were performed with the IBM SPSS 23.0 statistical package program (IBM Corp., Armonk, NY, USA). A two-sided p<0.05 was considered significant.

## RESULTS

The average age of the patients included in the study was 61.4±12.6 years. A total of 821 (54.8%) of the patients were male. When the demographic data of the patients were examined, it was determined that the patients who developed CIN were older (58.6±12.1 vs. 64.1±13.0, p<0.001) and were predominantly male [673 (54.1%) vs. 148 (57.8%), p=0.004]. Diabetes mellitus (DM), hypertension (HT), and heart failure (HF) were found to be more common in patients who developed CIN (p=0.001, p<0.001, and p=0.011, respectively). Other demographic data were found to be similar (Table 1).

**Table 1:** Baseline characteristics, laboratory results of all study patients, and patients with and without contrast-induced nephropathy.

		Non-CIN group, n=1242	CIN group, n=256	p-value
Demographics				
	Age, years	58.6±12.1	64.1±13.0	**<0.001**
	Male gender, n (%)	673 (54.1)	148 (57.8)	**0.004**
	Diabetes mellitus, n (%)	381 (30.8)	160 (62.7)	**0.001**
	Hypertension, n (%)	545 (44.0)	165 (64.5)	**<0.001**
	Hyperlipidemia, n (%)	758 (61.0)	169 (66.0)	0.138
	CAD, n (%)	474 (38.2)	108 (42.7)	0.203
	HF, n (%)	920 (74.1)	209 (81.6)	**0.011**
	Smoking, n (%)	381 (30.7)	81 (31.8)	0.211
	BMI, kg/m^2^	27.9±7.5	27.6±5.6	0.819
On admission, clinical characteristics				
	Systolic blood pressure, mmHg	135.7±39.6	135.9±36.1	0.340
	Heart rate, per minute	79.6±19.2	82.9±22.1	0.449
	Left-ventricular ejection fraction (%)	42.9±10.5	40.3±10.6	0.542
	sPAP, mmHg	35.0±7.8	39.2±8.4	**0.004**
MI type				
	Anterior MI	300 (24.2)	114 (44.5)	**0.001**
	Inferior MI	405 (32.6)	56 (21.9)	
	NSTEMI	537 (43.2)	86 (33.6)	
Laboratory results				
	Hemoglobin, g/dL	14.1±4.9	13.8±5.7	**0.040**
	White blood cell count, cells/μL	10.5±4.9	10.8±5.6	0.732
	Lymphocyte count, 10^9^/L	1.6±0.3	1.5±0.2	**<0.001**
	Platelet count, cells/μL	260.7±82.6	251.5±89.1	0.207
	CRP, mg/L	7.5±3.2	12.4±2.8	**0.009**
	Albumin, g/dL	3.7±0.5	3.2±0.6	**<0.001**
	Admission blood glucose, mg/dL	161.8±85.2	187.6±96.7	**0.001**
	Baseline creatinine, mg/dL	1.2±0.9	1.3±1.0	0.076
	Peak creatinine, mg/dL	1.4±1.1	1.8±1.2	**0.003**
	Peak creatinine kinase–myocardial band, ng/mL	70.8±21.2	117.6±18.8	0.390
	Peak troponin, ng/L	10562±531	11538±413	0.574
	NT-proBNP, pg/dL	2056±787	2935±766	**0.013**
	Total cholesterol, mg/dL	185.3±23.9	171.1±22.4	0.223
	TG, mg/dL	180.8±28.5	182.9±23.6	0.071
	HDL, mg/dL	39.1±12.5	39.9±16.8	0.109
	LDL, mg/dL	126.0±36.3	122.1±33.8	0.273
Angiographic and clinical data				
	Multi-vessel stenosis (>50%), n (%)	360 (29.0)	61 (23.8)	0.109
	LAD as the infarct-related artery, n (%)	608 (49.0)	163 (63.7)	**0.001**
	Contrast volume, mL	274.1±61.0	273.2±58.2	0.764
	Need for dialysis, n (%)	0 (0)	30 (11.7)	**<0.001**
	Length of hospital stay, days	6.4±5.1	8.8±4.6	**0.012**
	In-hospital mortality	34 (2.7)	24 (9.4)	**<0.001**
	OPS	0.84±0.25	2.2±0.32	**<0.001**
	GPS	0.42±0.15	1.46±0.13	**0.025**
	PNI	44.9±4.7	39.1±6.4	**0.001**
	SII	981.2±114.4	1177.9±111.8	**0.008**

CIN: contrast-induced nephropathy; CAD: coronary artery disease; HF: heart failure; BMI: body mass index; sPAP: systolic pulmonary artery pressure; MI: myocardial infarction; CRP: C-reactive protein; NT-proBNP: N-terminal prohormone brain natriuretic peptide; TG: triglycerides; HDL: high-density lipoprotein; LDL: low-density lipoprotein; LAD: left anterior descending artery; OPS: Osaka prognostic score; GPS: Glasgow prognostic score; PNI: prognostic nutritional index; SII: systemic immune-inflammation index. Bold values indicate statistically significant values.

**Figure 1:**
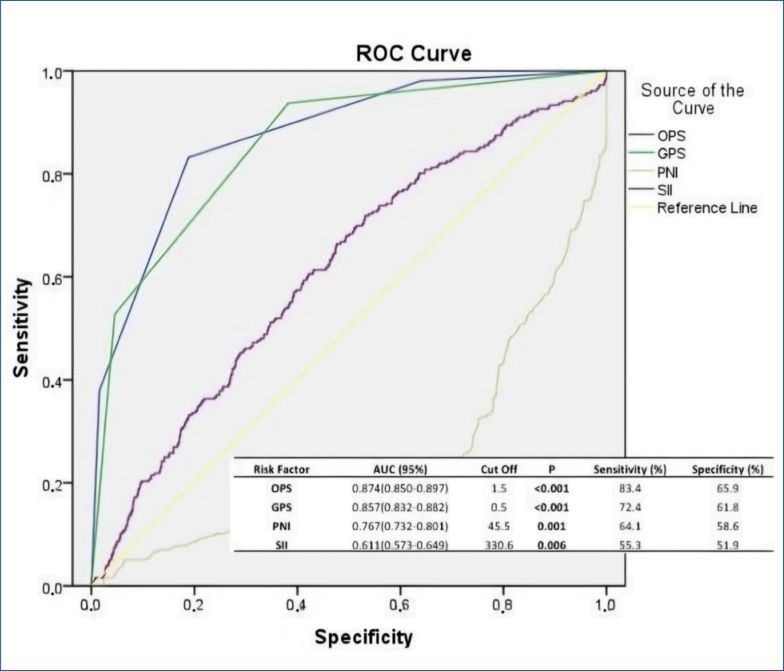
Receiver operating characteristics for Osaka prognostic score, Glasgow prognostic score, prognostic nutritional index, and systemic immune-inflammatory index.

When laboratory data were examined, in patients who developed CIN, hemoglobin, TLC, albumin, and PNI values were lower (p=0.040, p<0.001, p<0.001, and p=0.001, respectively). Systolic pulmonary artery pressure (sPAP), CRP, peak creatinine, N-terminal prohormone brain natriuretic peptide (NT-proBNP), OPS, GPS, and SII values were found to be higher (p=0.004, p = 0.009, p=0.003, p=0.013, p<0.001, p=0.025, and p=0.008, respectively) (Table 1). According to the univariate regression analysis, age, male gender, DM, HT, HF, sPAP, anterior MI, hemoglobin count, TLC, CRP, albumin, glucose, NT-proBNP, left anterior descending (LAD) as the infarct-related artery, OPS, GPS, PNI, and SII (p<0.001, p=0.001, p=0.001, p<0.001, p=0.011, p=0.003, p=0.001, p<0.001, p<0.001, p<0.001, p=0.001, p=0.001, p<0.001, p<0.001, p<0.001, p=0.004, p<0.001, and p=0.001, respectively) were found to be good prognostic factors in predicting CIN; as a result of multivariate analysis, age, DM, sPAP, anterior MI, hemoglobin count, TLC, CRP, albumin, Nt-proBNP, OPS, and SII were found to be independent risk factors for the development of CIN (p=0.001, p=0.001, p=0.001, p=0.001, p=0.046, p<0.001, p<0.001, p=0.001, p=0.001, p<0.001, and p=0.001) (Table 2). To evaluate the significance of OPS in predicting CIN as a result of ROC analysis, the AUC was 0.874 (95%CI: 0.850–0.897, p<0.001) and the optimal cutoff value was 1.5 (83.4% sensitivity and 65.9% specificity) (Figure 1).

**Table 2:** Univariate and multivariate analyses for the predictor of contrast-induced nephropathy.

	Univariate analysis	p-value	Multivariate analysis	p-value
	OR (95%CI)		OR (95%CI)	
Age	1.059 (1.047–1.070)	**<0.001**	1.046 (1.025–1.067)	**0.001**
Male gender	2.406 (1.817–3.186)	**0.001**	1.010 (0.610–1.1671)	0.970
Diabetes mellitus	3.793 (2.2863–5.024)	**0.001**	2.605 (1.612–4.210)	**0.001**
Hypertension	2.3112 (1.749–3.058)	**<0.001**	1.615 (0.985–2.650)	0.058
HF	1.556 (1.107–2.189)	**0.011**	1.659 (0.957–2.874)	0.071
sPAP	1.056 (1.041–1.072)	**0.003**	1.050 (1.026–1.074)	**0.001**
Anterior MI	3.008 (1.652–5.476)	**0.001**	2.521 (1.908–3.331)	**0.001**
Hemoglobin count	0.822 (0.774–0.873)	**<0.001**	0.913 (0.834–0.998)	**0.046**
Lymphocyte count	0.050 (0.024–0.103)	**<0.001**	0.109 (0.018–0.653)	**<0.001**
CRP	1.544 (1.465–1.628)	**<0.001**	1.479 (1.339–1.634)	**<0.001**
Albumin	0.167 (0.127–0.220)	**0.001**	0.256 (0.142–0.462)	**0.001**
Admission blood glucose	1.003 (1.002–1.004)	**0.001**	0.999 (0.997–1.002)	0.642
NT-proBNP	1.001 (1.001–1.002)	**<0.001**	1.001 (1.001–1.002)	**0.001**
LAD as the infarct-related artery	1.828 (1.384–2.413)	**<0.001**	0.942 (0.545–1.629)	0.832
OPS	8.128 (6.349–9.405)	**<0.001**	2.161 (1.101–4.241)	**<0.001**
GPS	5.441 (3.972–8.676)	**0.004**	1.098 (0.449–2.686)	0.838
PNI	0.825 (0.802–0.848)	**<0.001**	0.806 (0.782–1.004)	0.569
SII	1.002 (1.001–1.003)	**0.001**	1.001 (1.001–1.002)	**0.001**

CIN: contrast-induced nephropathy; DM: diabetes mellitus; HF: heart failure; sPAB: systolic pulmonary artery pressure; MI: myocardial infarction; CRP: C-reactive protein; NT-ProBNP: N-terminal brain natriuretic peptide; TG: triglycerides; HDL: high-density lipoprotein; LAD: left anterior descending artery; OPS: Osaka prognostic score; GPS: Glasgow prognostic score; PNI: prognostic nutritional index. Bold values indicate statistically significant values.

## DISCUSSION

This is the first study to examine the relationship between OPS and CIN in the current literature. As a result of our study, OPS was found to be an independent predictor of CIN development in patients undergoing PCI after ACS.

As a result of previous studies, it is known that patients with decreased nutritional status and increased inflammatory activity have worse cardiac outcomes, especially CIN^6,8,10^. In our study, consistent with the literature, CIN was more common in patients with high OPS, GPS, and SII and low PNI. However, as a result of our study, CIN was also found to be more common in patients with advanced age, male individuals, DM, and HT. It is known that especially with increasing age, systemic inflammatory imbalance and activity increase more, and nutritional status weakens^14,15^. This may be an additional parameter explaining why more CIN develops in patients with advanced age, which we found as a result of our study. Again, the fact that we found more CIN in male subjects is compatible with the literature and can be explained by the higher inflammatory response and lower nutritional status in men^16,17^. As another result of our study, CIN was higher in patients with DM and HT. Although DM and HT are direct risk factors for CIN, increased inflammation and decreased nutritional status, which are among the multifactorial etiologies of CIN, may have contributed to the development of CIN^18,19^. In our study, CIN was more common in patients with low hemoglobin levels. This finding has been shown many times before in the literature. Increased inflammation and poor nutritional status in patients with low hemoglobin values may also have played a role in the development of CIN^20^.

Our study focuses on OPS, a combination of several parameters of CIN development's most well-known physiopathological components. These are CRP, albumin, and TLC, respectively. All of these components reflect the systemic inflammatory response^21^. Increased inflammatory response is also known to increase the development of CIN. OPS also has components that reflect nutritional status. Decreased nutritional status increases the development of CIN and also induces systemic inflammation^7,8^. Thus, using only OPS can obtain information about the patient's nutritional and systemic inflammatory status. These components, known to increase the development of CIN individually, can give more accurate results with a single score. It can easily increase the prediction of CIN development. Furthermore, a single score can also reveal the patient's preoperative nutritional status and systemic inflammation. In our study, nutritional and inflammatory parameters such as GPS, PNI, and SII were evaluated in addition to OPS. OPS proved to be a superior parameter in predicting the development of CIN by having a higher AUC area as a result of ROC analysis. This superiority of OPS over GPS may be due to the additional inclusion of TLC. Indeed, a direct association between TLC and CIN is known. OPS thus reflects the systemic increased inflammatory state better than GPS. PNI includes only albumin and TLC values. It does not contain CRP as in OPS. As a result of this situation, PNI, which has a more nutritional aspect, may neglect to reflect systemic inflammation a little more. The superiority of OPS in showing CIN in our study may be because it better shows increased systemic inflammation due to CRP. According to Ma et al.'s study, SII has recently been found to be a trendy CIN indicator^22^. However, the SII contains only simple blood parameters and provides no information on nutritional status. Therefore, it needs to be improved in one aspect compared with OPS. In our study, OPS was a better predictive parameter than SII.

The main limitations of our study are that it was retrospective and covered a limited geographical area. The fact that our study was single-centered is another area for improvement. In addition, long-term follow-up was not performed in these patients, laboratory values at presentation and at the time of CIN development were used as spots, and long-term results are unknown. Another limitation is that many scoring systems are known to predict the development of CIN, and these scoring systems and parameters were not used in our study. Future randomized controlled trials are needed to confirm the results of our study.

## CONCLUSION

Our study found OPS as a parameter that may predict the development of CIN in patients undergoing PCI after ACS. OPS is an indicator that is relatively easy to calculate and its components are readily available in any healthcare institution. By using the OPS, patients with an exceptionally high risk for CIN development can be identified and treatment can be personalized to reduce the risk of CIN development.
